# *CRY1* Gene Polymorphism and Racing Performance of Homing Pigeons

**DOI:** 10.3390/ani11092632

**Published:** 2021-09-07

**Authors:** Andrzej Dybus, Hanna Kulig, Yu-Hsiang Yu, Ruben Lanckriet, Witold Proskura, Yeong-Hsiang Cheng

**Affiliations:** 1 Department of Genetics, West Pomeranian University of Technology, 70-311 Szczecin, Poland; hkulig@zut.edu.pl; 2 Department of Biotechnology and Animal Science, National Ilan University, Yilan 26047, Taiwan; yuyh@niu.edu.tw (Y.-H.Y.); yhcheng@ems.niu.edu.tw (Y.-H.C.); 3 PiGen vof, Keiberg 31, B-8552 Moen, Belgium; rubenlanckriet@telenet.be; 4 Faculty of Biotechnology and Animal Husbandry, West Pomeranian University of Technology in Szczecin, 71-270 Szczecin, Poland; witoldproskura@gmail.com

**Keywords:** cryptochromes, *CRY1* gene, homing pigeons, genetic markers, racing performance

## Abstract

**Simple Summary:**

The aim of this study was to detect polymorphism in cryptochrome 1 (*CRY1*) gene and to estimate associations between genotypes and racing performance in homing pigeons. The *AG* to *TT* change in intron seven of *CRY1* gene was detected and determined in 129 pigeons. A statistically significant relationship between genotypes and the results of racing was found. The results indicate the possibility of using this polymorphism as a genetic marker in pigeon breeding.

**Abstract:**

Cryptochromes (CRY) are the family of proteins proposed as the putative magnetoreceptor molecules. In birds, among others in pigeons, CRY1 is widely expressed in a retina. Homing pigeons are known for their navigational abilities, and pigeon racing is a popular sport. So, the purpose of this study was to analyze the variability of the nucleotide sequence of the homing pigeon *CRY1* gene, spanning the region coding the two amino acids W320 and W374 of Trp-triad, and estimate the relationship between genotypes and the racing performance. Investigations were carried out on 129 pigeons. Analysis of sequencing results indicated the *AG* to *TT* change within the seventh intron of *CRY1* gene. Genotypes were determined by the forced PCR-RFLP method. The influence of detected polymorphism on the results of racing pigeons in 100–400 km flights was shown. The *AG/TT* individuals achieved significantly higher (*p* ≤ 0.05) mean values of ace points (AP) than the *AG/AG* ones. Regarding the detected nucleotide change localization, the polymorphism may be involved in *CRY1* gene expression modulation. The *AG* to *TT* change in *CRY1* gene may be considered as a potential genetic marker of racing performance in homing pigeons.

## 1. Introduction

The phenomenon of magnetoreception in animals has fascinated the scientific world for many years. The ability of different animal species to use information from the geomagnetic field for navigation is well known, both in long-distance migratory animals (crab salmon, arctic tern) and in those who make local wanderings (bees, bats, pigeons) [1,2,3]. Behavioral evidence for the presence of the magnetic sense has been obtained, but the underlying molecular mechanisms remain under investigation. Most of the research conducted in this area focuses on the search for magnetic sensory cells based on the concept of light-dependent radical pairs and the concept of a system that would use biogenic magnetite [4,5,6]. There is also a known hypothesis that animals have the ability to detect a geomagnetic field by electromagnetic induction in the semicircular canals of the inner ear [7].

Birds are believed to have the ability to use the direction of the magnetic field lines as a “compass”, and the mentioned above radical pairs located in the eyes may play a key role. Moreover, it seems that birds may be sensitive to changes in the strength of the Earth’s magnetic field as part of the navigational “map”, and that magnetite-based receptors located in the upper beak could be involved in this ability [8,9,10]. Current trends in magnetoreception research assume the existence of several proteins that can act as magnetoreceptors, and one of the main candidates seems to be cryptochromes (Cry) [3,11,12]. Cryptochromes are commonly expressed in a variety of animal tissues, including the retina. These proteins are closely related to photolyases, belonging to flavoproteins, which are involved in the repair of DNA damaged by UV radiation [12,13]. However, cryptochromes do not show photolysis activity, but are involved in various blue light-dependent pathways as found in some organisms [14]. Some of these proteins have been shown to be involved in the regulation of circadian activity. Moreover, it is suggested that they also mediate the magnetic “compass” mechanism in birds [3,12]. Cryptochromes are believed to be candidates that meet the assumptions for the radical pair magnetoreceptor [15].

Four proteins from the cryptochrome family have been identified in the retina of birds: Cryptochrome 1a (Cry1a), Cryptochrome 1b (Cry1b), Cryptochrome 2 (Cry2), and Cryptochrome 4 (Cry4) [12,16,17]. Cry1a and Cry1b are alternative splicing variants differing in their C-terminal region. In migratory birds, including racing pigeons, Cry1a has been detected in the outer segments of UV/V cones. Cry1b has been detected in ganglion cells, displaced ganglion cells, and inner segments of photoreceptors [12]. The Cry2 protein has been localized in photoreceptors and ganglion cells, and Cry4–in the outer segments of longwave-sensitive single cones and of the double cones [3,18,19]. It is now believed that the Cry4 and Cry1a proteins are likely to be receptor molecules for detecting directions. Cry4 is supported by its constant expression in the retina [18]. Cry1a, despite variable expression that may indicate a greater contribution to the circadian rhythm, is considered a serious candidate for a magnetoreceptor [18,19]. Since pigeons’ navigational abilities undoubtedly affect flight quality, they can also affect racing results. Hence, the genes encoding Cry proteins seem to be an interesting object of research in this area.

In pigeons, polymorphic sites in selected genes were analyzed in relation with racing performance. Within the genes studied in this respect, there are, among others, genes encoding creatine kinase M-Type (*CKM*), lactate dehydrogenase A (*LDHA*), feather keratin (*F-KER*), and dopamine receptor D4 (*DRD4*) [20,21,22,23,24]. A relationship was found between g.129456C > T SNP in the *DRD4* gene and pigeon racing results for short distance [21] and between g.710T > C polymorphism of the feather keratin gene and long-distance races [22]. Moreover, *LDHA* microsatellite (TTTAT)_3–5_ polymorphism was significantly associated with deviated EBV values of longer total race distance [23]. There were also genomic studies aimed at a broad analysis of the molecular basis of sport performance [25,26] and in the case of racing pigeons, also spatial orientation and navigational performance [27,28]. The whole genomic studies carried out by Gazda et al. [27] and Shao et al. [28] led the authors to suggest that the traits enabling fast flight, endurance, and precise navigation are shaped by many genes, their activity, and regulation. The authors also pointed to some candidate genes that may underlie racing performance, with the most important being the *CASK* (calcium/calmodulin-dependent serine protein kinase) and *GSR* (glutathione-disulfide reductase) genes.

The aim of this study was to analyze the variability of the nucleotide sequence of the racing pigeon *CRY1* gene within the region coding the amino acids W320 and W374 of Trp-triad (two of three amino acids crucial for cryptochrome function in magnetoreception) and analysis of association between the polymorphism(s) detected and racing performance of homing pigeons.

## 2. Materials and Methods

### 2.1. Study Approval

This study was carried out in strict accordance with the recommendations of the National Ethics Committee on Animal Experimentation. The protocol was approved by Local Ethics Committee for Animal Testing of the West Pomeranian University of Technology in Szczecin (Protocol Number: 36/2012).

### 2.2. Animals, DNA Analyses

A total of 129 pigeons (127 homing pigeons, one utility pigeon of King breed and one rock pigeon, *Columba livia*) were included in the study. Whole blood samples were collected from the metatarsal vein into collection tubes (with K_3_EDTA). DNA was isolated using the salting-out method (Master Pure^TM^ DNA Purification Kit for Blood Version II, Epicentre Biotechnologies). The procedure of DNA purification was modified to avian blood and only 5 μL of whole blood was used in the first step of DNA extraction.

The following PCR primers were designed to produce a 672-base pairs (bp) fragment of the *CRY1* gene (a part of exon 7 and intron 7), spanning the codons for W320 and W374 amino acids of Trp-triad, using Primer3 (primer3.ut.ee) software and NW_004973449 sequence (Gene ID: 102088263):

CRY1-F: 5′-GGCAACTAACAATCCACGGT-3′
CRY1-R: 5′-TTGGGCAAAGGGACAGAAAC-3′

PCR mixture contained ~100 ng of genomic DNA, 12 pmol of each primer, 1× PCR buffer, 1.5 mM MgCl_2_, 0.2 mM dNTP mix, and 0.4 units Taq-polymerase in a total volume of 15 µL. The following cycles were applied: denaturation at 94 °C for 5 min, followed by 30 cycles at 94 °C for 30 s, primer annealing at 57 °C for 30 s, PCR product synthesis at 72 °C for 50 s, and final extension at 72 °C for 5 min. At the first stage of the study (i.e., DNA polymorphism detection), PCR products from six unrelated individuals (four racing pigeons—three Belgian ace pigeons for short, middle, and long distance via. Ruben Lanckriet, PiGenvof, Belgium and one racing pigeon via. Andrzej Dybus from West Pomeranian University Loft; one utility pigeon—King breed from a local breeder, Szczecin Poland; and one rock pigeon via. Prof. Eberhard Haase, Kiel, Germany) were sequenced by external service (Genomed, Warsaw, Poland).

The Chromas software (https://technelysium.com.au/wp/chromas/, accessed on 7 September 2021) was used for analysis of DNA sequencing results. The second pair of primers was designed for genotyping the pigeons included in the association study (123 pigeons). The forced PCR-RFLP method was used, with the following primers:

CRY-*Mlu*CI-F: 5′-AGAAACATTGGTTACTCTTATAGTTA*A-3′
CRY-*Mlu*CI-R: 5′-AACATTGGGCAAAGGGACAG-3′

In forward primer, one nucleotide modification (A* instead of complementary G) was designed to create the restriction motif for *Mlu*CI restriction enzyme in the 176 bp amplification product and enable genotyping of the *AG/TT* polymorphism (detected in previous DNA sequencing). PCR mixture contained ~100 ng of genomic DNA, 10 pmol of each primer, 1× PCR buffer, 1.5 mM MgCl_2_, 0.2 mM dNTP mix, and 0.3 units Taq polymerase in a total volume of 15 µL. The following cycles were applied: denaturation at 94 °C for 5 min, followed by 30 cycles at 94 °C for 20 s, primer annealing at 59 °C for 30 s, PCR product synthesis at 72 °C for 30 s, and final extension at 72 °C for 5 min. Amplicons were digested with 4 units of *Mlu*CI restriction endonuclease (NEB). The digestion products were separated by horizontal electrophoresis through a 3% agarose gels (PRONA) in 1× TBE and stained with ethidium bromide.

### 2.3. Statistical Analysis

A total of 123 racing pigeons (63 males and 60 females) were included in the association study. All pigeons were raced in full racing season (14 races) using the total widowhood method (males and females). The data for analysis consisted of the results achieved by individuals that participated in short (≤400 km) and long (≥500 km) distances. The birds came from one racing club (two top ranked breeders) in Lubusz Province of Poland. A total of 1209 race records were used in statistical analyses. Each pigeon that participated in the race was assigned 0 to 100 ace points (AP) system. The ace points are awarded to individuals that completed a given race (20% of starting pigeons won prizes). The winning pigeon is always awarded by 100 AP, calculated by the following formula [20]:

(1) $$AP=\frac{\left(a-b+1\right)}{a}\times 100$$

where:

*AP* is the ace points value
*a* is the number of pigeons on the prize list (20% of participated)
*b* is the position of a pigeon in a race

Associations between AP and *CRY1* gene polymorphism were analyzed with the following mixed model using lmekin package for R software:

(2) y=μ+g+s+h+k+ps+pp+k+i+a+e

where: *y*—analyzed trait; *µ*—overall mean; *g*—effect of genotype (CRY1*^AG/AG^*, CRY1*^AG/TT^*, CRY1*^TT/TT^*); *s*—gender effect (male, female); *h*—breeder effect (A, B); *ps*—weather at the start effect (sunny, changeable); *pp*—weather at the end effect (sunny, changeable, rainy, windy, cloudy); *k*—race category effect (short, long); *i*—effect of individual accounting for repeated observations (1–123); *a*—a random polygenic component account for all known pedigree relationships (3 generations); *e*—random error.

Pedigree data were handled using Pedigree Viewer 6.5b. The additive relationship matrix was based on a three-generation pedigree using the kinship2 R package.

## 3. Results

**Figure 1:**
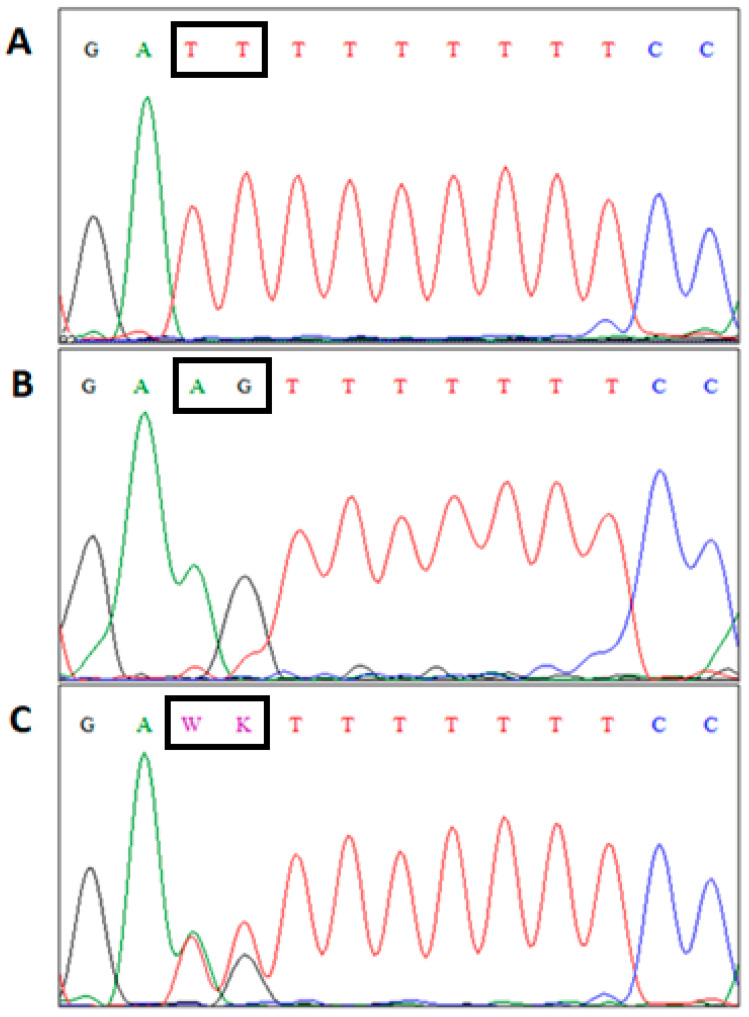
The results of sequencing analysis of *CRY1* gene. (**A**)/*CRY1^TT/TT^* genotype, (**B**)/*CRY1^AG/AG^*, (**C**)/*–CRY1^AG/TT^*; W = A or T; K = G or T.

Analysis of sequencing results (Chromas v.2.31) indicated the polymorphic sites (*AG* to *TT* change) in the analyzed amplicons that was located in intron seven of *CRY1* gene (Figure 1).

In the course of forced PCR-RFLP, the following DNA restriction fragments were observed for the *CRY1* gene AG to TT polymorphism (Gene ID: 102088263; g.31975-31976, *AG* > *TT*): 151 and 25 bp for the *CRY1^TT/TT^* genotype, 176, 151 and 25 bp for the *CRY1^AG/TT^* and 176 bp (no digestion) for *CRY1^AG/AG^* ones. The frequencies of genotypes and alleles of the polymorphism are presented in Table 1. It was shown that the *AG/AG* genotype was most frequent in analyzed pigeons (0.87), but the *TT/TT* genotype frequency was only 0.02. Nevertheless, the distribution of genotypes was in agreement with HWE expectation (Table 1).

Mean values of ace points in relation to the *CRY1* genotypes and the race category are given in the Table 2. The influence of the *AG/TT* polymorphism in *CRY1* gene on the racing pigeons’ performance in short distance races (100–400 km) was shown. Statistical analysis revealed that the *AG/TT* pigeons were characterized by significantly higher mean value of ace points (AP) than the *AG/AG* individuals (34.02 vs. 27.01, *p* ≤ 0.05). The highest values of APs in *CRY1-AG/TT* genotype group were reported for all races (33.79) and long-distance races (33.31), but the difference between the groups were not statistically significant (*p* = 0.11 and *p* = 0.19).

## 4. Discussion

The discovery of the homing ability in pigeons opened new possibilities that extend beyond that of a messenger. Racing pigeons is very popular, not only for recreational purposes but also as a prestigious and lucrative sport; many so-called “one loft races” are annually organized, with large monetary prizes for winners [29]. Homing pigeons, known for their outstanding navigational abilities, are an excellent research model for a better understanding of the phenomenon of magnetoreception. Initially, these capabilities were used to transmit information, e.g., from military and merchant expeditions. Currently, homing pigeons provide breeders with sports emotions by taking part in competitions [30]. Over the years, selection was carried out to improve athletic performance, such as speed, and flight endurance, which translated into racing results. An attempt to explain the genetic basis of the variability of these traits has become the subject of research conducted on racing pigeons.

The Earth’s magnetic field intensity, inclination angle, and magnetic declination can help many animals to determine their position and helps in migratory navigation [31,32]. Homing pigeons can also use a magnetic map over shorter distances [33]. Cryptochrome proteins use light energy to form long-lived radical pairs [4,14] and enable these proteins to be magnetically sensitive [34,35]. Du et al. [11] used the (recombinant) CRY1 protein of *Columba livia* (clCRY1) to investigate the pigeon magnetoreception. The authors have observed magnetic field effects on clCRY1 and evidence of cryptochromes acting as a magnetoreceptor in birds. In this study, we have found a new polymorphism (*AG* → *TT* change) in a sequence of intron 7 of *CRY1* gene of homing pigeon. This substitution is adjacent to the series of seven thymines located in 287–293 upstream to 5′ splice site and extends it by two more. In effect, it becomes one of the two longest thymine repeats in the seventh intron. This site appears to be extremely interesting as a pyrimidine rich region as it could potentially be related to splicing regulation. Reference may be made to a polypyrimidine tract (PyT) located between the branch point adenosine and the 3′ splice site. In vertebrate introns, the branchpoint sequence and a PyT are essential signals for efficient recognition of the 3′ splice site and for accurate splicing [36]. There are various ways in which introns affect gene expression, e.g., by affecting the transcription level, nuclear export, transcript stability, and translation efficiency [37,38,39,40]. However, for some enhancing introns, no such activity was found. Introns can differ in the presence and location of elements that positively or negatively affect expression. Chromatin modifications or secondary structures achieved by transcripts may also play a role in the activity of introns. Various ways in which introns affect the splicing process have also been documented [41].

Intronic regulatory elements (enhancers and silencers) are believed to be primarily recognized by splicing factors in a sequence-specific manner. Examples of splicing factors include the cytotoxic granule-associated RNA binding protein (TIA1) and TIA1-like 1. It was shown in vitro that TIA1 binds to U-rich sequences downstream of 5′ splice sites and is involved in recruiting U1snRNP to the 5′ splice sites [42]. TIA proteins can be antagonized by the pyrimidine pathway binding protein (PTB) due to their affinity for U-rich sequences [43]. The results of research by Gao et al. [44] indicate the importance of U-rich sequences downstream of the 5′ splicing site in regulating this process. Depending on the nucleotide composition, such motifs may be enhancer or silencer, and a single nucleotide change may transform an enhancer into a silencer or vice versa. Within introns, there are sequences similar to the consensus motifs of canonical splice sites, such as cryptic, non-canonical or pseudo splice sites. Cryptic splice sites may be used when a mutation occurs in the natural splice site [45]. It has been found that mutations located in splicing sites near intron boundaries, as well as within exon or intron RNA regulatory elements, are often associated with diseases, including neurodegenerative, neoplastic, and muscle atrophy [46].

Until now, studies of the *CRY1* gene polymorphism have been conducted in humans. Mutations located in this gene were associated with major depressive disorder (MDD), insomnia, and anxiety [47,48]. Our results are the first in this regard in a non-human species. The presented research showed differences in flight efficiency over short distance races in pigeons with different *CRY1* genotypes. The highest values of ace points were observed in individuals with the *AG/TT* genotype, and they were significantly higher than in homozygous *AG/AG* individuals. To sum up, the associations of polymorphism detected with racing performance of homing pigeons reported in this study may be also influenced by other (unknown) functional polymorphism in the *CRY1* gene, so further studies are needed to confirm our interesting observations.

## 5. Conclusions

In this study, the new detected intronic dinucleotide change in the *CRY1* gene was associated with racing performance in pigeons. The obtained research results lead to the suggestion that this dinucleotides polymorphism (minor allele frequency of 0.07) within the *CRY1* gene may be related with the modulation of its expression, and thus may have an impact on the pigeons’ results in the racing competitions. Nevertheless, more research is required to obtain evidence of its functionality, especially given that the current state of knowledge about the participation of specific intron elements, in addition to known motifs involved in splicing, in the processes related to the regulation of gene expression requires exploration.

## Figures and Tables

**Table 1:** Distribution of *CRY1* genotypes and alleles in analyzed pigeons.

Genotypes			Alleles		HWE	
*AG/AG*	*AG/TT*	*TT/TT*	*AG*	*TT*	Χ^2^	*p*
0.87 (*n* = 107)	0.11 (*n* = 14)	0.02 (*n* = 2)	0.93	0.07	3.1811	0.9268

*n*—number of individuals.

**Table 2:** Mean values of AP in relation to *CRY1* genotypes and the race category.

Genotype	All Races			Short Races (100–400 km)			Long Races (500–800 km)		
	RR	AP	SE	RR	AP	SE	RR	AP	SE
*AG/AG*	1017	25.61	1.07	666	27.01 *	1.36	351	22.94	1.73
*AG/TT*	176	33.79	2.86	118	34.02 *	3.55	58	33.31	4.89
*TT/TT*	16	28.16	9.45	12	30.90	11.70	4	19.94	15.83

RR—number of race records; AP—mean of ace points; SE—standard error of the mean; *—statistically significant differences at *p* ≤ 0.05.

## Data Availability

Data is contained within the article.
